# Novel approaches to the patient with massive hemoptysis

**DOI:** 10.3402/jchimp.v2i1.14784

**Published:** 2012-04-30

**Authors:** Scott Parrish, William Krimsky, Robert Browning, Mohamad Alabrash

**Affiliations:** 1 Walter Reed National Military Medical Center, Bethesda, MD, USA; 2 Franklin Square Hospital Center, Baltimore, MD, USA

**Keywords:** hemoptysis, fiberoptic bronchoscopy, endobronchial techniques

## Abstract

Massive hemoptysis is a life-threatening condition with a high mortality when treated conservatively. Several modalities have been described in the treatment of hemoptysis with varying results. Endobronchial therapy has traditionally been performed with rigid bronchoscopy. This requires both specialized training and equipment that is not readily available in many centers. The role of fiberoptic bronchoscopy (FOB) is unclear in these situations but is more widely accessible. We describe three cases of the successful treatment of hemoptysis with FOB. These patients were treated with a combination of techniques described previously in the literature; however, these methods failed to result in cessation of the bleeding. Therefore, we employed alternative strategies not described in the literature, using oxidized regenerated cellulose with FOB alone as well as in conjunction with endobronchial placement of vascular embolization coils. These additional techniques may offer other options when rigid bronchoscopy or other modalities are not readily available.

Established Facts

- Endobronchial treatment of hemoptysis has traditionally been performed with rigid bronchoscopy that is not readily available at many centers.

Novel Insights

- Fiberoptic placement of oxidized regenerated cellulose and vascular embolization coils are other modalities that may be performed in the peripheral airways to successfully to treat hemoptysis.

Massive hemoptysis is a life-threatening condition with a high mortality when treated conservatively. Interestingly, varying definitions in the literature exist of massive hemoptysis. These range from 100 to over 1000 mL in a 24-hour period (1). Malignant airway tumors, bronchitis, and bronchiectasis are typically the most common cause of massive hemoptysis but tuberculosis and lung abscesses among others have been reported (2). Tuberculosis remains an important cause of hemoptysis in the United States despite the decreased prevalence compared to more endemic countries (3).

As with the definition of massive hemoptysis, the approaches to treatment and therapy are variable. Several modalities involving arteriography have been described, including bronchial arterial embolization. Airway interventions include procedures performed with rigid and fiberoptic bronchoscopes and open surgical procedures. Rigid bronchoscopy is advantageous because it maintains a patent airway as well as allows larger instruments and suction catheters to be used. Unfortunately, a substantive percentage of these methods require highly specialized centers, including thoracic vascular radiology and rigid bronchoscopy. We present cases of massive hemoptysis treated with alternative methods using fiberoptic bronchoscopy (FOB) and tools available to bronchoscopists at most institutions.

## Case 1

A 63-year-old man with a history of hypertension, hyperlipidemia, type 2 diabetes mellitus, and cigarette smoking was admitted to the hospital following episodes of massive hemoptysis in the previous 24 hours. Admission laboratory work did not reveal a coagulopathy or thrombocytopenia. A chest radiograph demonstrated a radiodensity in the medial aspect of his left lower lung. A follow-up chest computed tomography (CT) scan was significant for a 4-cm spiculated cavitary mass in the superior segment of the left lower lobe that abutted the posterior mediastinum along with several subcentimeter nodules inferior to the lesion.

His presentation was highly suspicious for a primary bronchogenic carcinoma. The patient underwent bronchoscopy. The takeoff to the superior segment was significantly stenotic with induration and heaped-up mucosa. The FOB was advanced into the cavity. Forceps biopsies through the working channel of the bronchoscope were taken inside the cavity. This led to bleeding from the orifice of the superior segment that was initially controlled using a combination of recombinant thrombin and balloon bronchoplasty with a 4-Fr Fogarty balloon. Although the bleeding slowed, it, nevertheless, continued unabated.

At that point, two pieces of oxidized regenerated cellulose (ORC, Surgicell^®^, Johnson and Johnson's, London) approximately 15×15 mm were folded and placed into the jaws of a flexible biopsy forceps. The forceps were then withdrawn into the operating channel of the bronchoscope, and the scope was reinserted into the airways. The forceps were inserted into the cavity, and the ORC was deployed. No further samples were taken to prevent dislodgement of the ORC.

The pathology on the forceps biopsy was consistent with inflammatory changes. He then underwent thoracotomy 1 week later due to a continued concern for a malignancy. Prior to the thoracotomy, there were no further episodes of bleeding. The mass was noted to be quite fibrotic with involvement of a branch of the inferior pulmonary artery. Acid-fast bacilli were noted on pathology staining, and DNA probe was positive for *Mycobacterium tuberculosis*.

## Case 2

A 75-year-old man with a history of prior resection of a stage Ia non-small cell lung cancer (NSCLC) of the left upper lobe, renal transplant, prostate cancer, chronic obstructive lung disease, coronary artery disease, and atrial fibrillation presented with a 6-cm mass in the right middle lobe. He underwent FOB that yielded a diagnosis of NSCLC that appeared identical to his prior malignancy. The procedure was uncomplicated; he did well and was started chemotherapy for metastatic disease. Roughly, 4 weeks later, he presented with massive hemoptysis and was admitted to the hospital. The etiology of the bleeding was thought to be a consequence of recurrent bleeding from his tumor. A FOB was performed. Substantial amounts of bloody secretions were noted in the airways predominantly from the takeoff of the right middle lobe. After suctioning, there was continued oozing from the right middle lobe. Bleeding was initially controlled with recombinant thrombin and balloon tamponade with a 4-Fr Fogarty balloon. Electrocautery was used as well on visible areas of friable mucosa. Despite this, the bleeding continued.

The decision was then made to place ORC into the culprit airway lumen. Using the method outlined above in the first case, the ORC was placed into the lateral segment of the right middle lobe. While the bleeding slowed, there was some displacement of the ORC proximally so a Vortex embolism coil was placed into the lateral segment of the right middle lobe in an attempt to secure the ORC. This was accomplished without difficulty. At that point, hemostasis was achieved. He was then discharged without further incident and had no further bleeding and ultimately died from his underlying malignancy a few months later.

## Case 3

**Fig. 1:**
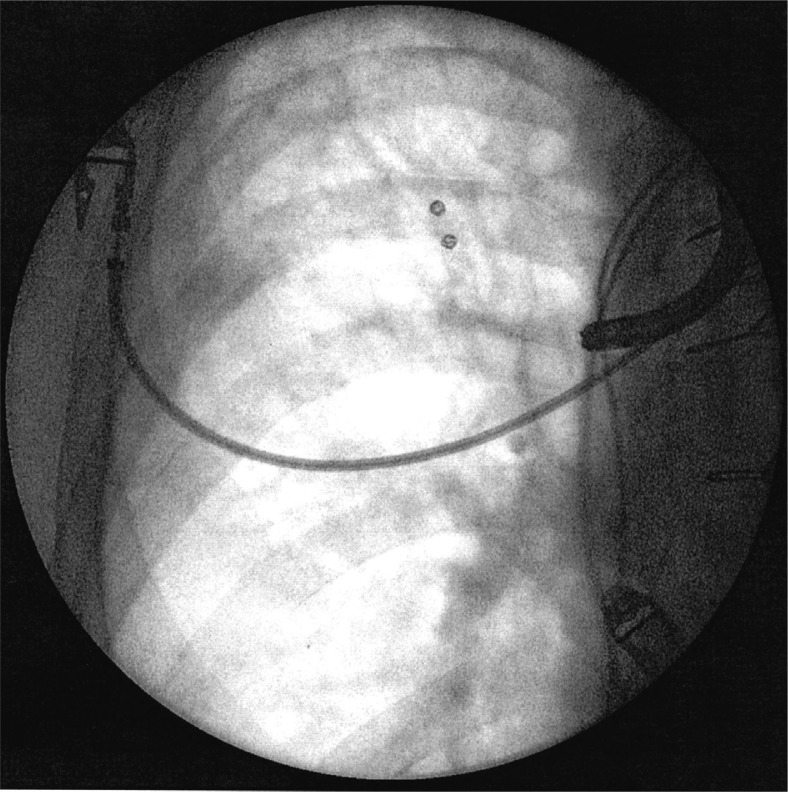
Fluoroscopic image of the patient in Case 3 demonstrating placement of two embolization coils in right upper lobe.

A 57-year-old gentleman with a history of aortic valve replacement on chronic Coumadin as well as history of pulmonary tuberculosis who presented to the hospital after coughing up copious amounts of blood for 2 days. His international normalized ratio was supratherapeutic at 3.3, but the rest of his admission laboratory work was otherwise unremarkable. A CT scan revealed significant bilateral upper lobe emphysematous changes as well as right upper lobe bronchiectasis (Fig. 1).

The patient was brought urgently to the bronchoscopy suite. FOB revealed significant bleeding from the apical segment of the right upper lobe. The airways were aggressively suctioned, and an embolism coil was initially placed in the subsegment to slow the bleeding. This was followed by ORC placement as described above. Another coil was then placed to secure the ORC. There was no evidence of active bleeding, and the patient was able to be brought safely to the ICU and later discharged. As of the writing of this manuscript, there have been no further complaints of hemoptysis.

## Discussion

There are multiple modalities for controlling bleeding in the lung. For life-threatening hemoptysis, the airway should be protected by placing the bleeding side in a recumbent position and protecting the non-bleeding side with consideration of a mainstem placement of a single lumen endotracheal tube, double lumen endotracheal tube, or bronchial blocker (4). Bronchial artery embolization (BAE) is often used to control the bleeding. However, while immediate control of bleeding is quite good, rebleeding is fairly common and has been reported in over 50% of patients in some series (5). Surgery is typically reserved for cases refractory to BAE. Patients who undergo surgical resection during active bleeding have a high rate of morbidity and mortality (6).

There are variety of techniques that can be employed with FOB in the setting of hemoptysis. These include topical application of agents such as epinephrine, thrombin or fibrinogen-thrombin, iced saline lavage, endobronchial blockade with a balloon, and, in some cases, the use of laser therapy or electrocautery (7). Despite this, the role of FOB in massive hemoptysis or hemoptysis in general remains unclear. While early versus late bronchoscopy has a higher yield for localizing, the source of bleeding FOB can be limited as a consequence of the inability to see the bleeding site due to blood filling the airways and limited suction capability (1, 8).

Some of the techniques used in this series have been described before. Tsukamoto et al. described the method for using thrombin and fibrinogen–thrombin infusion using a fiberoptic bronchoscope with good results (9). The use of a Fogarty balloon to occlude the segmental or subsegmental bronchus leading to the bleeding site is also a described modality and typically is left in place for 24 to 48 hours with careful monitoring of the patient for rebleeding after the balloon is deflated (10). Valipour et al. reported the use of ORC in 57 patients with massive hemoptysis, albeit from a central airway source, achieving immediate hemostasis in 98% of patients. Approximately, 10.6% of those patients had recurrence of bleeding. The authors used rigid bronchoscopy initially and then a FOB through the rigid scope to place the ORC into the central airways (11). Nogueira et al. reported two cases where application of ORC to the bronchi using rigid bronchoscopy was successful in the cessation of hemoptysis (12). These techniques are similar in that they control the bleeding in the central airways.

However, the case series presented here describes two unique approaches to management of this often life-threatening problem and offers alternative approaches to control airway bleeding in cases where rigid bronchoscopy or vascular radiology is not readily available. ORC dissolves to form a gel matrix that promotes clot formation when in contact with blood. Vascular coils cause mechanical disruption of blood flow, which leads to thrombogenesis. In our review of the literature, no reports exist specifically addressing placing ORC directly into a bleeding cavity only using FOB as we described above. In the first case, bleeding was both initially controlled using a combination of balloon bronchoplasty, topical thrombin, and placement of ORC using only FOB delivered outside of the central airways. The last two cases expand on this approach with the delivery of a vascular embolization coil placed directly into the culprit bronchial lumen using it not only to potentially secure the ORC but also, as with the third case, the coil itself was used to initially slow the bleeding followed by ORC and an additional embolization coil. To our knowledge, this is the first report on endobronchial placement of an embolization coil to help control bleeding.

While the numbers are obviously small, the clinical results suggest both efficacy and reproducibility. These techniques appear to be reasonable alternatives and complimentary to each other in the treatment of hemoptysis and can be used separately from or in conjunction with rigid bronchoscopy. Further investigation is needed to confirm these observations.
